# First Insight into Diversity of Minisatellite Loci in *Mycobacterium bovis*/*M. caprae* in Bulgaria

**DOI:** 10.3390/diagnostics13040771

**Published:** 2023-02-17

**Authors:** Daria Terentieva, Tanya Savova-Lalkovska, Albena Dimitrova, Magdalena Bonovska, Igor Mokrousov, Violeta Valcheva

**Affiliations:** 1 Laboratory of Molecular Epidemiology and Evolutionary Genetics, St. Petersburg Pasteur Institute, 197101 St. Petersburg, Russia; 2 National Reference Laboratory for Bovine Tuberculosis, National Diagnostic and Research Veterinary Medical Institute “Prof. Dr. G. Pavlov”, 1606 Sofia, Bulgaria; 3 Department of Infectious Microbiology, The Stephan Angeloff Institute of Microbiology, Bulgarian Academy of Sciences, 1113 Sofia, Bulgaria

**Keywords:** *Mycobacterium bovis*, *Mycobacterium caprae*, VNTR, genotyping, whole-genome sequencing, Bulgaria, bTB

## Abstract

The aim of this study was to assess the diversity of minisatellite VNTR loci in *Mycobacterium bovis*/*M. caprae* isolates in Bulgaria and view their position within global *M. bovis* diversity. Forty-three *M. bovis*/*M. caprae* isolates from cattle in different farms in Bulgaria were collected in 2015–2021 and typed in 13 VNTR loci. The *M. bovis* and *M. caprae* branches were clearly separated on the VNTR phylogenetic tree. The larger and more geographically dispersed *M. caprae* group was more diverse than *M. bovis* group was (HGI 0.67 vs. 0.60). Overall, six clusters were identified (from 2 to 19 isolates) and nine orphans (all loci-based HGI 0.79). Locus QUB3232 was the most discriminatory one (HGI 0.64). MIRU4 and MIRU40 were monomorphic, and MIRU26 was almost monomorphic. Four loci (ETRA, ETRB, Mtub21, and MIRU16) discriminated only between *M. bovis* and *M. caprae*. The comparison with published VNTR datasets from 11 countries showed both overall heterogeneity between the settings and predominantly local evolution of the clonal complexes. To conclude, six loci may be recommended for primary genotyping of *M. bovis*/*M. caprae* isolates in Bulgaria: ETRC, QUB11b, QUB11a, QUB26, QUB3232, and MIRU10 (HGI 0.77). VNTR typing based on a limited number of loci appears to be useful for primary bTB surveillance.

## 1. Introduction

Bovine tuberculosis (bTB) caused by either *Mycobacterium bovis* or *M. caprae* is an important zoonotic disease with a serious burden on global livestock production [1,2]. The informed knowledge of bTB epidemiology to implement bTB surveillance and control measures requires adequate and affordable tools to study its transmission. The primary goal is that these tools should be sufficiently discriminatory to trace particular clones and clonal clusters of the pathogen. However, the cost and ease of use are no less important for the low-resource areas, many of which are also areas with significant livestock production. It has long been believed that the *M. tuberculosis* complex has low genetic diversity, however, molecular studies in the last 10–15 years have clearly demonstrated genetic variation within the complex on the whole and its species/subspecies in particular. Whole genome sequencing (WGS) is the most informative tool in this regard, permitting us to discriminate at different levels of genetic relatedness and to confirm/delineate phylogenetic lineages within the *M. tuberculosis* complex (including *M. bovis* lineage) and smaller genetic groups and clonal complexes [3,4]. However, WGS remains expensive, especially for a large-scale analysis. Furthermore, wide implementation of somewhat sophisticated bioinformatics tools remains a challenge. In this view, the use of known genotyping tools may be helpful as a primary and affordable approach in primary epidemiological surveillance. Two such methods include spoligotyping and Variable Number of Tandem Repeats (VNTR) typing. The advantages and drawbacks of these methods are well known. The major problem with spoligotyping is its single-locus nature (hence, the non-independent evolution of the 43 spacers/characters) and low discrimination of closely-related local strains. Spoligotyping is useful for rapid strain differentiation under global analysis, but it does not allow tracing transmission when spoligotypes are dominant and/or widespread [4,5].

On the other hand, the typing of minisatellite VNTR loci , initially based on 6 Exact Tandem Repeats (ETR) loci [6], was further expanded, and the current approach to genotype human *M. tuberculosis* is based on 24 loci [7]. The generated digital profile is specific for a strain or group of closely related strains. However, the typing of 24 loci is cumbersome, and optimized reduced sets of loci have been searched for in different settings. The inclusion of additional hypervariable loci was required for some genetically homogeneous genotypes. For *M. bovis,* several typing schemes based on a reduced number of VNTR loci were proposed, while in other studies, a complete set of 24 loci complemented with even more loci were applied [8,9]. Based on an analysis of the long-term national collection, Hauer et al. [4] suggested that spoligotyping and VNTR typing used together are phylogenetically informative, predicting diverse *M. bovis* strain lineages at the national level under long-term surveillance. In Spain, the continuous use of VNTR typing for 20 years revealed that the largest cluster corresponds to an *M. bovis* strain that was mainly spread during the 1990s [10]. VNTR typing was useful to trace *M. bovis* transmission in Poland among different animal species and importation from the UK to Poland by infected alpacas [11,12,13]. In Switzerland, VNTR typing was informative to detect the re-emergence of an endemic strain in the western region of the country and the likely transborder penetration of the other strain from Austria [14].

In this study, we describe the 13-loci VNTR typing of *M. bovis*/*M. caprae* field isolates from cattle collected over 6 years in Bulgaria. We sought to assess the level of VNTR diversity of the studied isolates and to propose a combination of the most discriminatory VNTR loci that would be suitable for relatively simple epidemiological surveillance of bTB in Bulgaria, which could be implemented in the prospective studies to trace transmission and imported cases. In addition, we assessed the recently developed SAM-TB online tool [15] for WGS-based phylogenetic analysis and compared the discrimination results achieved by WGS and VNTR.

## 2. Materials and Methods

### 2.1. Bacterial Strains

Based on Bulgarian national regulations and National bTB Control Program that has been implemented since 2015, suspicious bTB samples were submitted to the National Reference Laboratory (National Diagnostic and Research Veterinary Medical Institute “Prof. Dr. G. Pavlov”, Sofia, Bulgaria) for confirmation of bTB diagnosis by bacteriological culturing and PCR. An analysis of the animal specimens in this study was therefore carried out within an official context, meaning that no animals were killed for the purposes of this research project, and ethical approval was not required.

The collection included all isolates available in the National Reference Laboratory for bTB. The Program for Monitoring and Control of Bovine Tuberculosis in Bulgaria includes annual, one-time intradermal tuberculin testing for bovine PPD tuberculin of all cattle over 42 days of age. Doubtfully PPD tuberculin-reacted animals undergo differential tuberculin testing with bovine and avian PPD tuberculin on day 42 after the first tuberculin test. Animals that positively react to regular and differential tuberculinization are sent for sanitary slaughter at isolation slaughterhouses. In 2015–2021, 71 TB-affected farms with 864 TB-positive animals were identified in 11 provinces across the country.

Diagnostic materials from the lymph nodes of slaughtered animals that responded positively or doubtfully to the tuberculin test were tested in the National Reference Laboratory for Tuberculosis in Animals (Sofia, Bulgaria). The samples were subjected to pathoanatomical examination for the presence of tuberculous lesions. Tissue suspensions were prepared for microbiological and PCR assays in accordance with World Organization for Animal Health protocols [2], as described previously [16]. Each sample was inoculated in parallel in MGIT tubes in Löwenstein–Jensen medium with pyruvate and in Stonebrink medium with pyruvate and PACT at 37 °C and 5% CO_2_. The smears were prepared from the cultures and were subjected to Ziehl–Neelsen staining.

DNA was extracted directly from tissue samples with the NucleoSpin^®^ Tissue kit (Macherey-Nagel GmbH & Co. KG, Dueren, Germany) and from cultured mycobacterial strains using the Seeplex MTB/NTM ACE kit (Seegene, Irvine, CA, USA). The latter kit was used to identify *M. tuberculosis* complex isolates. *M. bovis* (including *M. caprae*) isolates were identified using the *Mycobacterium bovis* amplification kit (Genekam Biotechnology AG, Duisburg, Germany). The identified *M bovis* isolates were further tested by RD4-PCR as described [17] to differentiate between *M. bovis sensu stricto* and *M. caprae*.

### 2.2. Genotyping

The isolates were previously tested by spoligotyping, which was followed by a comparison with SITVIT_WEB (http://www.pasteur-guadeloupe.fr:8081/SITVIT2/, accessed on 10 January 2023). This permitted us to assign SIT to the isolates, and consequently, to differentiate between *M. bovis* and *M. caprae* [16,18].

Genotyping of the 13 VNTR loci (Table 1) was performed using the primers described previously [6,7,19,20,21]. We used different kinds of ordinary or hot-start *Taq* polymerases: *rTaq* (GE Healthcare, Chicago, IL, USA), *TaqF* (Interlabservis, Moscow, Russia), Hot Start *Taq* (Sibenzyme, Novosibirsk, Russia), *Taq* (Sileks, Moscow, Russia), and different thermal cyclers (BioRad, Hercules, CA, USA C1000, T100, and TurboCycler, Blue-Ray, Biotech). The PCR products were separated in 1.3–1.5% standard agarose gels; a 100 bp ladder (GE Healthcare), or Step100 ladder (Biolabmix), were used as a molecular weight markers. The reason for using different polymerases and thermocyclers was technical. PCR failure was observed for certain loci in some isolates. For this reason, we used different polymerases and PCR machines to repeat the PCR and obtain amplified products. The use of different polymerases and thermocyclers did not influence the size of the amplified bands.

The MIRU-VNTRplus online tool (https://www.miru-vntrplus.org/MIRU/index.faces, accessed on 20 December 2022). was used to build a VNTR-based UPGMA dendrogram and minimum spanning tree (MST). VNTR alleles were treated as discrete variables. Under phylogenetic analysis, pairwise distances were calculated based on the loci that were successfully typed in both isolates, i.e., missing data were not considered.

The Hunter Gaston index (HGI) was used to assess the diversity of the individual loci and discrimination by different VNTR combinations and was calculated at http://insilico.ehu.es/mini_tools/discriminatory_power/index.php (accessed on 30 December 2022).

### 2.3. Bioinformatics Analysis of WGS Data

For this analysis, we used publicly available WGS data of 34 *M. bovis*/*M. caprae* isolates from Bulgaria (out of 43 isolates included in this study). These data were previously deposited at the NCBI Sequence Read Archive under accession number PRJNA785819. In this study, we performed a new bioinformatics analysis of these WGS data. The DNA of the other nine, more recent isolates was of insufficient quality and could not be used for WGS.

The SAM-TB online tool (https://samtb.uni-medica.com/index, accessed on 20 December 2022) [15] was used for SNP calling and phylogenetic analysis. The method implemented in the SAM-TB online tool is detailed in [15]. It included fastq file processing, alignment to the reference genome, and SNP calling, while PE and PPE repetitive regions and some other regions were excluded. This resulted in a shorter list of variations (compared to the PHYRESSE tool (not shown)), hence, there was less distance between isolates since some SNPs were not considered. Finally, the obtained data were analyzed using the same SAM-TB tool to create a concatenated fasta file and build the dendrogram.

The generated concatenated fasta file was also used for network building using the PHYLOViZ tool (https://online.phyloviz.net/index, accessed on 24 December 2022).

## 3. Results and Discussion

### 3.1. VNTR Diversity and Phylogenetic Analysis

A total of 43 *M. bovis*/*M. caprae* isolates were included in this study. These isolates were recovered from cattle in different farms in Bulgaria from 2015 to 2021. Some of them were previously characterized by spoligotyping and WGS [16,18]. All of the isolates were subjected to genotyping using minisatellite VNTR loci mainly based on the recommended eight loci scheme [9]. Whereas the reproducibility of PCR results was confirmed by repeating some of the isolates at least twice, some loci showed poor amplification. In particular, permanent PCR failure in some isolates was observed for loci QUB26 and QUB3232, even after we repeated the experiment with different polymerases and different thermocyclers. For this reason, we additionally analyzed five more loci, including those of the discriminatory *M. tuberculosis* format [7]. Thus, 13 VNTR loci were typed in total. The HGI of single loci was calculated based on all of the tested isolates with the available PCR results (Table 1). The HGI was also calculated for the *M. caprae* subgroup (Table 2). Different loci demonstrated different levels of diversity in the total collection (Table 1). On the other hand, the diversity of the individual loci was much lower for some loci in the *M. caprae* subgroup, which reflected that the overall diversity of these loci (ETRA, ETRB, QUB26 Mtub21, and MIRU16) was mostly due to their divergence between *M. bovis* and *M. caprae*.

**Figure 1:**
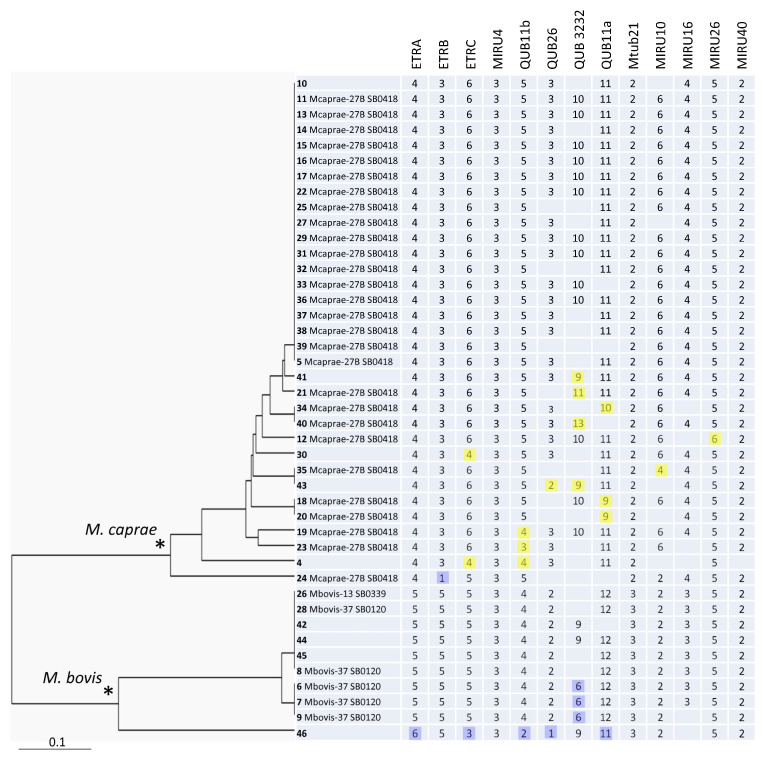
The 13-VNTR UPGMA tree of all 43 *M. bovis* isolates from Bulgaria. ID includes information on strain number (in bold), followed by the WGS-based group, and spoligotype number in Mbovis.org (if available). Minor alleles with regard to the main alleles in the branch (*M. bovis,* blue, or *M. caprae,* yellow) are highlighted. * Two main branches of *M. bovis* and *M. caprae* are marked by asterisks.

The phylogenetic analysis demonstrated a clear separation of the *M. bovis* and *M. caprae* branches on the 13-loci-based tree (Figure 1). The larger and more geographically dispersed *M. caprae* group included more profiles and appeared to be more diverse than the smaller *M. bovis* group did (HGI = 0.67 vs. 0.60). Six clusters and nine orphans were identified (from 2 to 19 isolates), and the HGI was 0.79 (Table 3).

The most discriminatory locus was QUB3232 (HGI = 0.64) although PCR failure was observed in some isolates. QUB3232 is termed a hypervariable locus and was not initially included in the 24 locus scheme of *M. tuberculosis* due to its uncertain reproducibility and performance on the whole [7]. It was shown that large PCR fragments (>1000 bp) were difficult to assign to alleles in a non-negligible proportion of *M. tuberculosis* strains. Nonetheless, the value of this locus was reconsidered [21], and it was included in the updated consensus scheme for subtyping the Beijing genotype using hypervariable loci [20]. Similarly, we believe that since QUB3232 still provides the best discrimination in *M. bovis*/*M. caprae*, it should be retained in VNTR typing formats for bTB surveillance.

Similar to QUB3232, high-copy alleles were also observed in the QUB11a (2163a) locus, whose analysis also requires longer gel run and expanded 100-bp ladders with fragment sizes of above 1000 bp. Practically, the use QUB3232 and QUB11a may be improved by the inclusion of control isolates with known alleles along with molecular weight markers with expanded fragment sizes to better detect large bands.

An overall separation between *M. caprae* and *M. bovis* can be concordantly seen in different loci (ETRA-B-C, Mtub21, QUB26, MIRU10, and MIRU16) (Figure 1). MIRU4 and MIRU40 were monomorphic. Of the five other loci, MIRU26 was almost monomorphic one, while ETRA, ETRB, Mtub21, and MIRU16 discriminated only between the *M. bovis* and *M. caprae* branches (Figure 1). We suggest that these seven loci can be excluded from the discriminatory optimized set of loci in our setting. Taking into consideration the locus diversity, the added value of discrimination, and the redundancy of the particular loci, six loci may be ultimately recommended for the primary genotyping of *M. bovis/M. caprae* isolates in Bulgaria: ETRC, QUB11b, QUB11a, QUB26, QUB3232, and MIRU10. The HGI of these 6 loci together was very close to the HGI based on 13 loci, 0.77 vs. 0.79, as were the clustering characteristics (Table 3). The same six loci (except for QUB26) are also the most variable ones among the *M. caprae* isolates alone. On the other hand, if a subdivision between *M. bovis* and *M. caprae* is required as a primary task, this can be achieved by the analysis of loci ETRC, MIRU10 (from the above six-loci list), Mtub21, or MIRU16.

There are different formats used for *M. bovis* typing with different levels of success/performance. Several studies followed the 24-loci format of Supply et al. [7], although some of the loci were low-variable or monomorphic ones. In some other studies, this format was slightly modified by the exclusion or inclusion of certain loci. In Mozambique, the inclusion of three hypervariable VNTR loci (3232, 3336, and QUB11a/2163a) resulted in minor changes only in the overall dendrogram, and a small increase in HGI from 0.87 (24 loci) to 0.93 (27 loci) [8].

The comparison with the data from other studies [8,9,22,23,24,25,26,27,28] regarding the diversity of these loci showed both overall heterogeneity between the settings and some similarities for certain loci (Table 4). Caution in interpretation is needed since HGI in different countries depends on the sample size, i.e., small samples may be biased towards under- or overestimation (Table 4). Overall, the analysis of the diversity of all of the loci in this study and some other studies from different continents revealed low or null diversity of some loci, but this varied between countries/settings.

MIRU4 (ETRD) was previously shown in an analysis of large European collections to be the least discriminatory one. Indeed, the same result was observed in this and other studies (HGI ranged from 0 to 0.24). However, this locus was moderately discriminatory for the Chinese Sika deer population (HGI 0.61). On the other hand, Mtub21 showed contrasting values from 0.36 to 0.50 (Bulgaria and Turkey, respectively) and almost no diversity (Brazil and Mozambique). Additionally, heterogeneous and contrasting HGI values were observed for ETRC and QUB11b. ETRB was discriminatory (0.40–0.64), except for in one setting (Mozambique, 0.06).

It may be noted that some loci may be homogeneous within a setting but the alleles may differ between the settings. For example, MIRU10 has two repeats in Brazil and Tunisia (cattle) and in Poland (bison), eight in China (deer), two in *M. bovis,* and six in *M. caprae* in Bulgaria (cattle, this study). This may depend on the animal host population and the country since mycobacterial/animal coadaptation could be different in different animal species and, respectively, *M. bovis* subspecies.

The eight-loci format initially used in our study was previously described [4,9] and included six loci of the Venomyc European consortium complemented with ETRC and QUB26. In Tunisia, the use of these eight loci resulted in a high HGI: 0.98. The useful point about 24 loci [7] is that they are well optimized and validated in terms of performance. On the other hand, other loci may achieve higher discrimination, but PCR failure is not rare and should be dealt with. In the Mozambique study, PCR failure was observed in many strains in the three additional loci [8]. Since the 27-loci format is cumbersome, inclusion in the analysis of the even more loci is even less practical for long-term permanent surveillance and typing of large collections. We refer to the study in Switzerland, where 49 loci were used to investigate two recent outbreaks: 24 loci of the phylogenetic format of Supply et al. and 25 other loci, including 6 microsatellite loci [14].

Similar to our study, the analysis of the global and long-term VNTR dataset from France, Spain, Portugal, Italy, Northern Ireland, and Belgium identified QUB3232 and MIRU4 as the most and the least discriminative loci, respectively [4]. In contrast, MIRU4 had a better discrimination capacity in human *M. tuberculosis* [7] and *M. caprae* [29,30]. Furthermore, the above study by Hauer et al. [4] revealed marked differences in the diversity of other MIRU-VNTR loci, which depends on the locally dominant clonal complexes. This highlights the necessity to use country-optimized sets of the most informative loci that have been proven to be the most discriminatory for the locally prevalent clonal complexes.

### 3.2. Global M. bovis VNTR Diversity

We collected VNTR data on the 13 loci included in this study from 12 countries [12,13,22,23,24,25,26,27,28,31,32] and built the dendrogram (Figure S1). Only the main types from the respective countries are included in the final tree: 271 types comprising 916 isolates, including 7 types shared by single isolates from different countries, as revealed in the preliminary analysis. The information on the VNTR alleles of the types is shown in Table S1.

The sample appears to be representative, although not exhaustive, and not all of the 13 loci were analyzed in those studies. The list of the included studies is not exhaustive. The criteria for inclusion were rather to have a general idea of global diversity and include studies from different continents. The profiles with double alleles were excluded. The data were missing in some studies due to PCR failure for certain loci, sometimes in the same isolates [8,24,29], and this could be due to the low quality of the DNA. Similar to our study, large alleles of QUB11a were amplified and were not precisely deciphered, for example, they were presented as >12 [22]. We did not include very large collections in our analysis because they spanned a very long time and would present a disproportionally large diversity, e.g., a French study of isolates collected from 1978 to 2013 [4]. We did not include a large dataset from African countries compiled by Machado et al. [8] because only four loci (ETR: A, B, C, and D) overlapped with those used in our study. Ultimately, we sought to determine differences in local *M. bovis/M. caprae* populations to find signature loci for rapid primary assessments and possible discrimination between the local vs. imported strains.

The observed lack of discrimination between *M. bovis* and *M. caprae* shows the lack of the differentiation capacity of the selected loci on a global scale (Figure S1). This could be due to the limited number of VNTR loci included in the analysis. Perhaps if a larger dataset of 20–27 loci was targeted, better and clearer discrimination would be achieved.

The global VNTR tree showed almost no types shared by the strains from different countries (Figure S1). There are a few exceptions shown in Figure S2: (i) the type of isolates from Algeria (cattle) and China (deer) with six identical loci; (ii) two pairs of isolates from China and Tunisia, China and Portugal with only three overlapped loci; (iii) two pairs of isolates from Brazil and Portugal (five loci); (iv) a pair of isolates from Portugal and Turkey (six loci); (v) a pair of isolates from Mexico and Mozambique (seven loci). However, these clusters were likely false since they were based on identity in only a few loci, seven loci at most. Furthermore, the identical isolates in clusters shown in Figure S2 were not only from distant countries, but different host species, which makes the possibility of transmission even less likely. A homoplastic evolution of VNTR loci leading to similar or identical profiles of the otherwise unrelated strains may impact the analysis in the case of a small number of available characters/loci.

Some *sensu lato* clusters (>90% similarity of profiles) included isolates from different countries, for example: (i) from Algeria and Turkey; (ii) from Brazil and Portugal; (iii) from Mexico, Portugal, and Tunisia. Interestingly, an isolate from Algeria was identical in 11 out of 12 loci with *M. bovis* field strain from a cattle outbreak in Switzerland (17 identical field isolates plus one archival sample). A closer look at all of the typed loci in these two studies [14,27] revealed that these isolates are identical in 16 out of 19 loci, including hypervariable loci QUB3232 and QUB11a. Whether this similarity reflects a true, albeit distant, relatedness or convergent evolution is unknown.

On the whole, most of the branches on the global tree (Figure S1) included isolates from one country each (Bulgaria, Italy, Portugal, Poland, Tunisia, or Mexico), which may reflect a mainly local evolution of the endemic clones. In the case of Poland, an additional factor enhancing the separate location of the isolates could be another host species (bison). One should keep in mind that some collections represent long-term studies, leading to higher heterogeneity and presence in different parts of the tree. The Chinese sample was the most diverse one and was mostly located apart from the other branches, which may reflect the geographic diversity of the sampled areas across China and the diversity of the host species *Cervus nippon*.

A closer look at the position of the Bulgarian isolates within the global tree revealed that 9 out of 10 *M. bovis* isolates were located distantly from their closest neighbors, while one isolate differed in two loci (out of ten) from an isolate from cattle in Turkey (Figure S2). One *M. caprae* isolate from Bulgaria differed in one out of seven loci from isolates from Portugal of various origins (cattle, wild boar, and red deer). However, other *M. caprae* isolates from Bulgaria are grouped together and distantly from other branches on the tree (Figure S1).

In two instances, we observe a relatedness between the isolates from Tunisia (human) and Algeria (cattle) (Figure S2), which may hypothetically reflect transmission events. Otherwise, other human *M. bovis* isolates from Tunisia are grouped separately on the dendrogram (Figure S1).

The historical and recent links between the countries might help us to interpret the observed diversity or similarity. Portugal, Brazil, and Mozambique are Portuguese-speaking countries with continued human migration between them and are also connected epidemiologically. For example, *M. tuberculosis* strains circulate between the countries [33]. Whether these countries have a meat or cattle trade exchange is unknown. The available information suggests a negative answer at least as far as the trade of live cattle is concerned [34,35,36]. Our findings based on VNTR data from different countries are similar to conclusions by Machado et al. [8] in eastern Africa, which appears to show high diversity, suggesting long-term evolution of *M*. *bovis* in that region. The diversity of *M*. *bovis* in Africa does not seem to be a function of the recent importation of animals, but is probably maintained within each particular region by constant reinfection from reservoir animals.

### 3.3. WGS Analysis and Comparison with VNTR Typing

**Figure 2:**
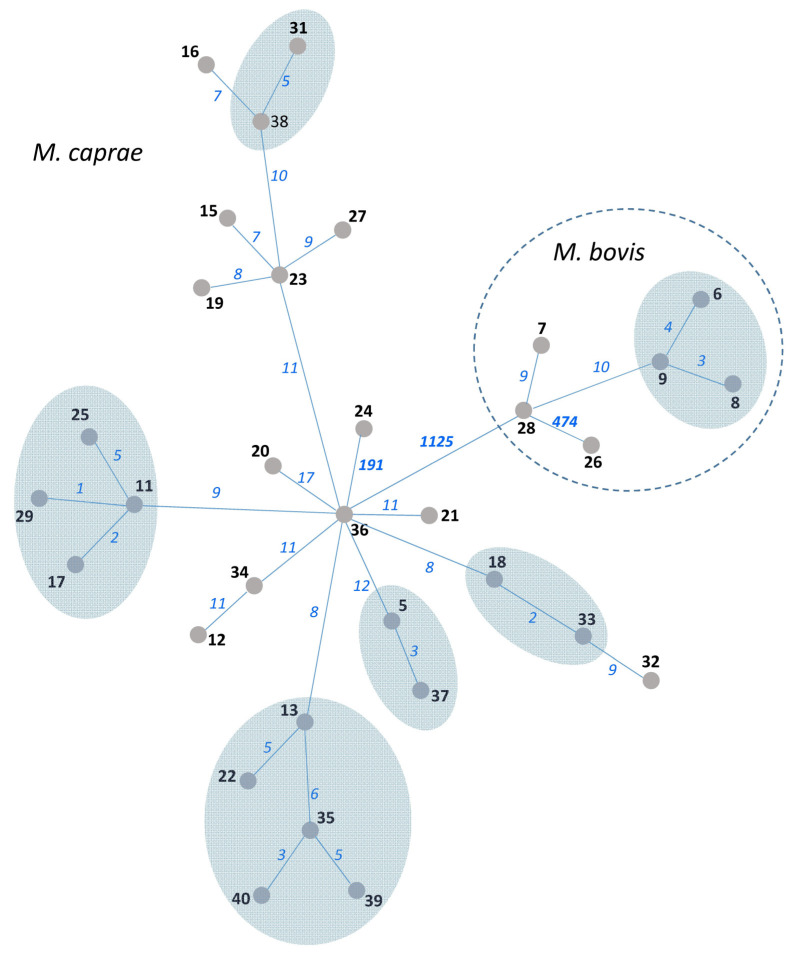
MST of 34 *M. bovis*/*M. caprae* isolates from Bulgaria based on WGS data. Isolate numbers are in bold. Numbers of SNPs separating neighboring nodes are on connecting branches and in italic. Branch lengths do not correlate with distance. SNP-based clusters of recent transmission with a 6-SNP threshold are shown by blue-shaded areas.

WGS data were available for 34 out of 43 Bulgarian *M. bovis*/*M. caprae* isolates used in this study. These data were previously published and analyzed using an in-house method in the National Veterinary Services Laboratories, USDA, Ames, IA, USA [16]. In the present study, we submitted these fastq files to the new analysis by an automatic method using the SAM-TB online tool, and the resulting MST is shown in Figure 2. The six SNPs threshold was applied to delineate clusters (i.e., isolates likely linked through recent transmission). Thus, six clusters can be identified on the WGS tree, and these encompass 18 isolates in total. They include five clusters of *M. caprae* isolates and one cluster of *M. bovis* isolates.

The 34 isolates with available VNTR and WGS data were used to compare three methods: 13-loci VNTR (HGI = 0.71 (Figure S3)), spoligotyping (two SIT), and WGS (all isolates were different but some differed only in one to three SNPs (Figure 2)). Spoligotyping is definitely not the method for high-resolution typing. In turn, VNTR typing greatly depends on the number and nature of VNTR loci used. The addition of the auxiliary loci can improve discrimination, but it would make this method even more time-consuming. While the discriminatory capacity of the 13 loci VNTR typing in this study was moderate, nonetheless, we believe that the VNTR method can be used for phylogenetic discrimination and surveillance (but not for tracing recent transmission, for which a high-resolution WGS approach is required).

It is known that currently available online tools to analyze *M. tuberculosis* raw WGS data (fastq files) perform SNP calling to assign lineage and to detect drug resistance mutations, but, to the best of our knowledge, they do not allow the phylogenetic analysis of the user strains. In this view, we note the utility of SAM-TB for the automatic phylogenetic analysis of the WGS data. The useful point about SAM-TB is that it does not only generate a list of variations of the isolate under study compared to the reference genome, but it permits us to perform a phylogenetic analysis in an automatic and user-friendly way. A negative point is that some diversity located in the excluded repetitive regions is not considered, and possible mechanisms of mycobacterial adaptation linked to PE/PPE variation may be missed.

## 4. Conclusions

To conclude, this study provided the first insight into the VNTR diversity of animal *M. bovis*/*M. caprae* isolates in Bulgaria. The larger and more geographically dispersed *M. caprae* group was more diverse than the *M. bovis* group was (HGI 0.67 vs. 0.60). A subdivision between *M. bovis* and *M. caprae* may be achieved by conducting an analysis of loci ETRC, MIRU10, Mtub21, or MIRU16. Six loci may be recommended for the primary genotyping of *M. bovis/M. caprae* isolates in Bulgaria: ETRC, QUB11b, QUB11a, QUB26, QUB3232, and MIRU10. We would not exclude locus QUB3232 from the *M. bovis*/*M. caprae* typing, but we mindful of the difficulty of its use in amplification and allele calling in case of large fragments with 10 and more repeat units. Practically, the use of QUB3232 and QUB11a may be improved by the inclusion of control isolates with known alleles, along with molecular weight markers on the large fragment scale, to better detect large bands.

Whereas WGS is the most useful and informative high-resolution approach to identify the transmission and emergence of *M. bovis/M. caprae* strains, it is not yet applicable for large-scale, routine use for bTB epidemiology in many countries, and not only low-resource countries. Since WGS is hardly feasible at a large scale, easier and less expensive approaches, such as VNTR, may be used for the time being. A strong local structure and clonal evolution of *M. bovis/M. caprae* imply that local VNTR sets should be complemented in some instances with intercountry VNTR sets to trace strains imported mostly through animal or animal products trade or through the transborder spread. A consensus VNTR format for *M. bovis* typing remains to be agreed upon, to be flexible and discriminatory, and to achieve different objectives depending on the local situation, i.e., giving more focus on local transmission within the country versus more emphasis on the detection of imported strains.

## Figures and Tables

**Table 1:** Diversity of VNTR loci in *M. bovis*/*M. caprae* (all collection) from Bulgaria.

Locus	No. of Allele Profiles	No. of Clusters	Cluster Size	HGI
ETRA/2165	3	2	9, 33	0.38
ETRB/2461	3	2	10, 32	0.40
ETRC/577	4	3	2, 10, 30	0.47
MIRU04/580	1	1	43	0
QUB11b/2163b	4	2	11, 30	0.46
QUB26/4052	3	2	10, 24	0.46
QUB3232/3232	5	3	3, 5, 13	0.64
QUB11a/2163a	4	3	2, 8, 27	0.46
Mtub21/1955	2	2	10, 33	0.36
MIRU10/960	3	2	11, 26	0.46
MIRU16/1644	2	2	8, 29	0.35
MIRU26/2996	2	1	41	0.05
MIRU40/802	1	1	42	0

**Table 2:** Diversity of VNTR loci in *M. caprae* isolates from Bulgaria.

Locus	No. of Allele Profiles	No. of Clusters	Cluster Size	HGI
ETRA/2165	1	1	33	0
ETRB/2461	2	1	32	0.06
ETRC/577	3	2	2, 30	0.17
MIRU04/580	1	1	33	0
QUB11b/2163b	3	2	2, 30	0.17
QUB26/4052	2	1	24	0.08
QUB3232/3232	4	2	2, 13	0.42
QUB11a/2163a	3	2	2, 26	0.20
Mtub21/1955	1	1	33	0
MIRU10/960	3	1	26	0.14
MIRU16/1644	1	1	29	0
MIRU26/2996	2	1	31	0.06
MIRU40/802	1	1	33	0

**Table 3:** Clustering of *M. bovis*/*M. caprae* isolates based on the different number of loci.

All 13 Loci Together	No. of Isolates	No. of Allele Profiles	No. of Clusters	Cluster Size (Range)	No. of Clustered Isolates	HGI
All 43 isolates	43	15	6	2-19	34	0.79
*M. caprae*	33	12	4	2-19	25	0.67
*M. bovis*	10	3	2	3-6	9	0.60
All 43 isolates (6 loci *)	43	14	6	2-20	35	0.77
34 isolates with WGS data	33	10	5	2-18	28	0.71

* minimal set of 6 loci: ETRC, QUB11b, QUB11a, QUB26, QUB3232, and MIRU10.

**Table 4:** Diversity of 13 VNTR loci in *M. bovis* in this and other studies.

Setting, Host, Years, Sample Size (Reference)	Bulgaria Cattle 2015–2021, *n* = 43 (This Study)	Portugal, 2002–2016, Cattle *n* = 384, Red Deer *n* = 303, Wild Boar *n* = 261 [22]	Tunisia, Human 2013–2015, *n* = 110 [9]	China, Sika Deer, 2008–2012, *n* = 96 [28]	Brazil, Cattle *n* = 17 [25]	Mozambique, Cattle 2007–2013, *n* = 178 [8]	Turkey, 2018–2019, Cattle *n* = 76, Human *n* = 4 [24]	Mexico, Cattle, 2009–2010, *n* = 132 [23]	Italy, Cattle 2000–2006, *n* = 1560 [26]	Algeria, Cattle 2017, *n* = 59 [27]
ETRA/2165	0.38	0.67	0.70	0.61	0.69	0.37	0.69	0.5	0.47	0.76
ETRB/2461	0.40	0.57	0.71	-	0.57	0.06	0.36	0.5	0.64	0.58
ETRC/577	0.47	0.53	0.27	0	0.57	0.04	0.45		0	0.56
MIRU04/580	0	0.24	0.15	0.61	0	0		0.02	0	0.18
QUB11b/2163b	0.46	0.58	0.62	0.74	0.16	0.16	0.50	0.63	0.32	0.69
QUB26/4052	0.46		0.64	0.59	0.58	0.47	0.59	0.66	0.34	0.48
QUB3232/3232	0.64	0.41	0.56	-	-	0.51		0.79	0.51	0.72
QUB11a/2163a	0.46	0.40	0.62	-	-	0.47		0.5	0.37	0.69
Mtub21/1955	0.36		-	0.59	0.06	0.04	0.50			
MIRU10/960	0.46		-	0	0	0.13			0	0
MIRU16/1644	0.35		-	0.27	0.57	0.43			0.14	0
MIRU26/2996	0.05	0.27	-	0	0.06	0.45	0.17	0.29	0.35	0.05
MIRU40/802	0		-	0.50	0	0	0.17		0.03	0.05

## Data Availability

Data of this study are presented in the article and Supplementary Materials.

## Supplementary Materials

The following supporting information can be downloaded at: https://www.mdpi.com/article/10.3390/diagnostics13040771/s1, Table S1: VNTR datasets from different countries used for global VNTR dendrogram. Figure S1: Global VNTR-based UPGMA tree of *M. bovis/M. caprae* isolates. Figure S2: Parts of the global VNTR-based UPGMA tree of *M. bovis/M. caprae* isolates: clusters of identical isolates from different countries, and some pairs of related isolates discussed in the main text. Figure S3: 13-VNTR UPGMA tree of 34 *M. bovis* isolates from Bulgaria with available WGS data.
