# Supplementary material for: First Insight into Diversity of Minisatellite Loci in Mycobacterium bovis/M. caprae in Bulgaria
Source: Diagnostics (Basel). 2023 Feb 17;13(4):771. doi: 10.3390/diagnostics13040771 (PMC9955489; doi:10.3390/diagnostics13040771)

Figure S1. Global VNTR-based UPGMA tree of *M. bovis*/*M. caprae* isolates.

ID includes country of origin, number of isolates, host.

Loci within used 13-loci scheme are in bold.

Abbreviations used in type ID. BRA – Brazil, BG – Bulgaria, CHN – China, ITA – Italy, MEX – Mexico, MOZ – Mozambique, PT – Portugal, TUN – Tunisia, TUR – Turkey, PL – Poland, SW – Switzerland.

The tree is based on data in Table S1.

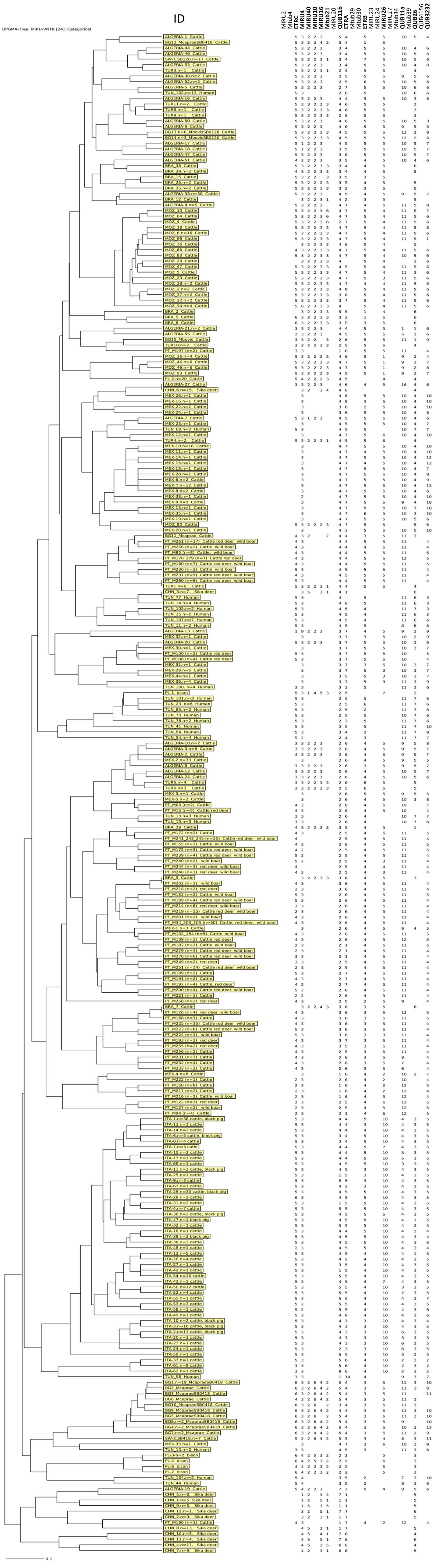

Supplement: Supplementary file 1 [file diagnostics-13-00771-s001.zip › Supplementary Figure S1.pdf]
