# Supplementary material for: First Insight into Diversity of Minisatellite Loci in Mycobacterium bovis/M. caprae in Bulgaria
Source: Diagnostics (Basel). 2023 Feb 17;13(4):771. doi: 10.3390/diagnostics13040771 (PMC9955489; doi:10.3390/diagnostics13040771)
Supplement: Supplementary file 1 [file diagnostics-13-00771-s001.zip › Supplementary Figure S2.pdf]

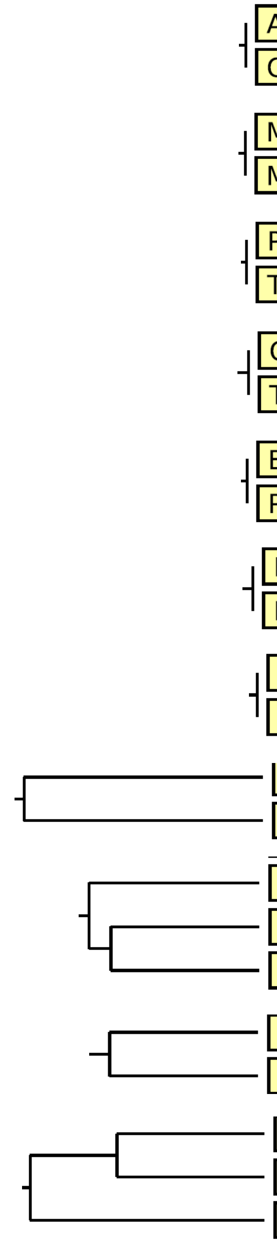

ID	MIRU2	Mtub4	**ETRC**	**MIRU4**	**MIRU40**	**MIRU10**	**MIRU16**	**Mtub21**	MIRU20	**QUB11b**	**ETRA**	Mtub29	Mtub30	**ETRB**	MIRU23	MIRU24	**MIRU26**	MIRU27	Mtub34	**QUB11a**	Mtub39	**QUB26**	QUB4156	**QUB3232**
ALGERIA-27 Cattle			3	3	2	2	3			4	6			4			5			16		4		6
CHN_6.n=15. Sika deer				3	2		3	4		4	6											4		
MEX-19.n=1 Cattle				3						4	7			5			5			10		5		6
MOZ_80 Cattle			5	3	2	2	2	3		4	7			5			5					5		6
PT_M260 (n=6) Cattle red deer wild boar			5	3						4	4			5			5			11				4
TUR1.n=6. Cattle			5	3	2	2	3	1		4	4			5			5					4		
CHN_3.n=7. Sika deer				3	5		3	1		4	1											6		
TUN_77 Human			5	3						4				5						11		6		3
BRA_9 Cattle				3	2	2	3	3		3	6			4			5					3		
PT_M202 (n=1) wild boar			4	3						3	6			4			5			11				4
BRA_7 Cattle				3	2	2	4	3		3	6			3			5					6		
PT_M126 (n=4) red deer wild boar			4	3						3	6			3			5			12				5
CHN_2.n=9. Sika deer				3	2		3	1		1	7											5		
PT_M190 (n=1) Cattle			4	3						1	7			4			2			12				4
BG15_Mbovis Cattle			3	3	2	2		3		2	6			5			5			11		1		9
TUR10.n=2. Cattle			3	3	2	2	3	3		2	5			5			5					4		
BG11_Mcaprae Cattle			4	3	2			2		4	4			3			5			11		3		
PT_M261 (n=37) Cattle red deer wild boar			4	3						4	4			5			5			11				4
PT_M266 (n=2) Cattle wild boar			4	3						4	4			4			5			11				4
ALGERIA-5 Cattle			5	3	2	2	3			2	7			5			5			10		6		6
TUN_102.n=13 Human			5	3						2	6			5						10		6		6
ALGERIA-7 Cattle			5	3	1	2	3			4	5			5			5			10		4		6
MEX-23.n=1 Cattle				3						4	5			5			5			10		4		7
TUN_68.n=2 Human			5	3						4	5			5						10		7		7

Figure S2. Parts of the global VNTR-based UPGMA tree of *M. bovis/M. caprae* isolates: clusters of identical isolates from different countries, and some pairs of related isolates discussed in the main text.

Loci within used 13-loci scheme are in bold. Abbreviations used in ID. BRA – Brazil, BG – Bulgaria, CHN – China, MEX – Mexico, MOZ – Mozambique, PT – Portugal, TUN – Tunisia, TUR – Turkey.
