# Supplementary material for: First Insight into Diversity of Minisatellite Loci in Mycobacterium bovis/M. caprae in Bulgaria
Source: Diagnostics (Basel). 2023 Feb 17;13(4):771. doi: 10.3390/diagnostics13040771 (PMC9955489; doi:10.3390/diagnostics13040771)
Supplement: Supplementary file 1 [file diagnostics-13-00771-s001.zip › Supplementary Figure S3.pdf]

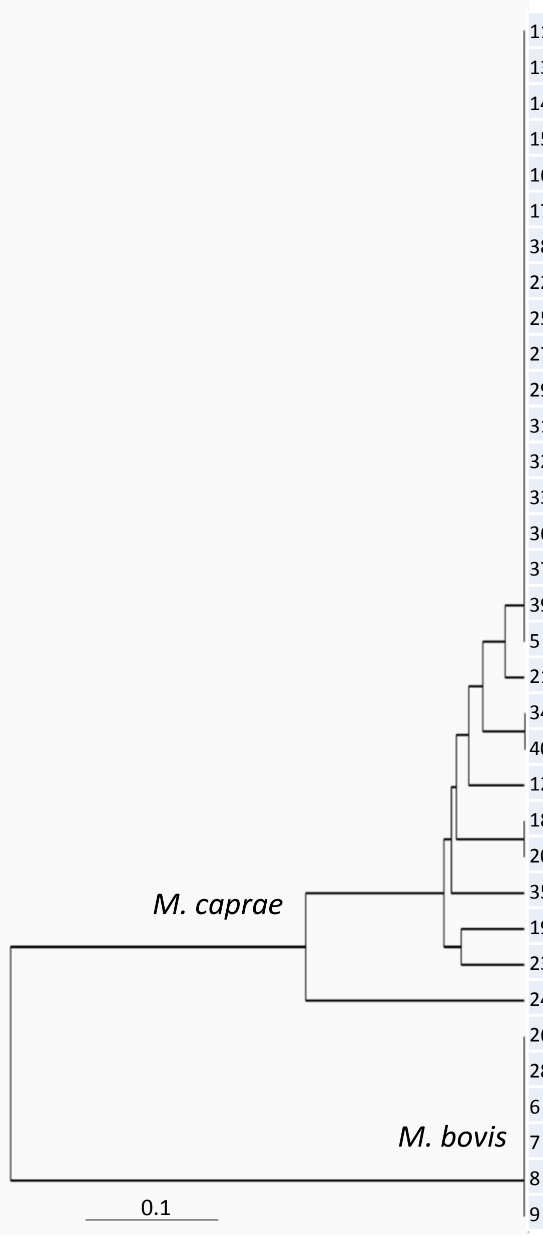

Isolate	ETRA	ETRB	ETRC	MIRU4	QUB11b	QUB26	QUB 3232	QUB11a	Mtub21	MIRU10	MIRU16	MIRU26	MIRU40
11 Mcaprae-27B SB0418	4	3	6	3	5	3	10	11	2	6	4	5	2
13 Mcaprae-27B SB0418	4	3	6	3	5	3	10	11	2	6	4	5	2
14 Mcaprae-27B SB0418	4	3	6	3	5	3		11	2	6	4	5	2
15 Mcaprae-27B SB0418	4	3	6	3	5	3	10	11	2	6	4	5	2
16 Mcaprae-27B SB0418	4	3	6	3	5	3	10	11	2	6	4	5	2
17 Mcaprae-27B SB0418	4	3	6	3	5	3	10	11	2	6	4	5	2
38 Mcaprae-27B SB0418	4	3	6	3	5	3		11	2	6	4	5	2
22 Mcaprae-27B SB0418	4	3	6	3	5	3	10	11	2	6	4	5	2
25 Mcaprae-27B SB0418	4	3	6	3	5			11	2	6	4	5	2
27 Mcaprae-27B SB0418	4	3	6	3	5	3		11	2		4	5	2
29 Mcaprae-27B SB0418	4	3	6	3	5	3	10	11	2	6	4	5	2
31 Mcaprae-27B SB0418	4	3	6	3	5	3	10	11	2	6	4	5	2
32 Mcaprae-27B SB0418	4	3	6	3	5			11	2	6	4	5	2
33 Mcaprae-27B SB0418	4	3	6	3	5	3	10		2	6	4	5	2
36 Mcaprae-27B SB0418	4	3	6	3	5	3	10	11	2	6	4	5	2
37 Mcaprae-27B SB0418	4	3	6	3	5	3		11	2	6	4	5	2
39 Mcaprae-27B SB0418	4	3	6	3	5				2	6	4	5	2
5 Mcaprae-27B SB0418	4	3	6	3	5	3		11	2	6	4	5	2
21 Mcaprae-27B SB0418	4	3	6	3	5		11	11	2	6	4	5	2
34 Mcaprae-27B SB0418	4	3	6	3	5	3		10	2	6		5	2
40 Mcaprae-27B SB0418	4	3	6	3	5	3	13		2	6	4	5	2
12 Mcaprae-27B SB0418	4	3	6	3	5	3	10	11	2	6		6	2
18 Mcaprae-27B SB0418	4	3	6	3	5		10	9	2	6	4	5	2
20 Mcaprae-27B SB0418	4	3	6	3	5			9	2		4	5	2
35 Mcaprae-27B SB0418	4	3	6	3	5			11	2	4	4		2
19 Mcaprae-27B SB0418	4	3	6	3	4	3	10	11	2	6	4	5	2
23 Mcaprae-27B SB0418	4	3	6	3	3	3		11	2	6		5	2
24 Mcaprae-27B SB0418	4	1	5	3	5				2	2	4	5	2
26 Mbovis-13 SB0339	5	5	5	3	4	2		12	3	2	3	5	2
28 Mbovis-37 SB0120	5	5	5	3	4	2		12	3	2	3	5	2
6 Mbovis-37 SB0120	5	5	5	3	4	2	6	12	3	2	3	5	2
7 Mbovis-37 SB0120	5	5	5	3	4	2	6	12	3	2	3	5	2
8 Mbovis-37 SB0120	5	5	5	3	4	2		12	3	2	3	5	2
9 Mbovis-37 SB0120	5	5	5	3	4	2	6	12	3	2		5	

Figure S3. The 13-VNTR-loci UPGMA tree of 34 *M. bovis* isolates from Bulgaria with available WGS data. The WGS-based tree of the same isolates is shown in Fig. 2.
ID includes information on strain number (in bold), followed by WGS-based group, and Spoligotype number in Mbovis.org.
